# Presentation of a new preemptive endoscopic treatment concept in duodenal interventions exemplified by an iatrogenic duodenal perforation after percutaneous transrenal nephrostomy. English version

**DOI:** 10.1007/s00104-025-02300-4

**Published:** 2025-05-20

**Authors:** V. Betz, A. Goerdt, R. Kiesow, J. Müller, B. Riefel, E. Scharsack, U. Zimmermann, M. Reeh, G. Loske

**Affiliations:** Klinik für Allgemein‑, Viszeral‑, Thorax- und Gefäßchirurgie, Kath. Marienkrankenhaus Hamburg gGmbH, Alfredstr. 9, 22087 Hamburg, Germany

Using a case study of an iatrogenic duodenal perforation after percutaneous transrenal nephrostomy, we show how a new preemptive endoscopic treatment concept can be used in addition to surgical therapy.

## Case presentation, diagnostic and operative procedure

A percutaneous transrenal nephrostomy was performed on an outpatient basis on a 65-year-old male patient due to unclear symptomatic grade 2 ectasia of the renal pelvicalyceal system (RPCS) on the right side with an unclear endoluminal space-occupying lesion with preexisting prostate cancer and multiple myeloma.

**Fig. 1:**
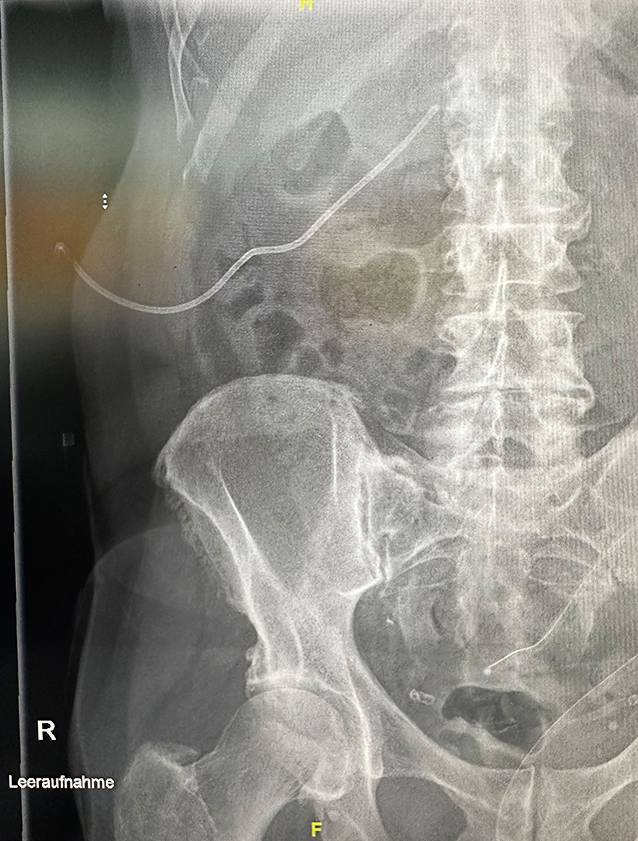
Nephrostomy catheter in place, plain X‑ray at the time of admission

After 5 days, the patient presented on an emergency basis due to the clinical suspicion of a misplaced nephrostomy catheter. Biliary secretions were draining out of the catheter. There was no pain or fever or macrohematuria. Laboratory chemistry tests did not show any elevated infection parameters. On ultrasound, grade 2–3 hydronephrosis was found in the right renal pelvicalyceal system, and the left kidney was unobstructed. A plain X‑ray in one plane showed the nephrostomy from the right side with the tip projecting into the renal pelvis at the level of the right transverse process of L1 (Fig. 1).

With clear clinical symptoms of a misplaced catheter, an emergency exploratory laparotomy was performed. After performing the Kocher maneuver, it was confirmed that the catheter tip had been moved from the immediately adjacent renal pelvis into the duodenum. The catheter was removed, and both the defect in the renal capsule and the duodenal defect were closed with 4‑0 polydioxanone sutures (MonoPlus®, B. Braun SE, Melsungen, Germany).

Due to the continued severe dilation of the right kidney and an elevated serum creatinine value, a new percutaneous transrenal nephrostomy on the right side was performed by our urology colleagues at this institution on the first postoperative day.

### Specifications

We assume that most readers will consider the sole surgical closure of the duodenal defect by suture and the usual supportive treatment measures, such as the administration of antibiotics and the provision of parenteral fluids, to be sufficient.

After the procedure, monitoring of the postoperative course follows, with checking of the infection parameters. One waits to see whether the clinical healing process is undisturbed and thus a sufficient closure of the defect can be assumed.

Interventions on the duodenum are often initially associated with impaired gastric passage. Therefore, in cases of an indicated emergency intervention, general anesthesia, and laparotomy, a passive gastroduodenal drainage tube is usually placed transnasally in the pre-, peri-, and postoperative periods. The position of the tube is digitally monitored during the operation and the tube is left in place as an overflow tube for the first postoperative phase after the procedure. In the absence of reflux, the drain is removed and a gradual return to a normal diet follows.

#### Question.

Which additional endoscopic treatment method could be used to supplement defect closure in the duodenum solely by surgery?

#### Answer.

Endoscopic negative pressure therapy (ENPT) in the duodenum can also be used as a preemptive measure for anastomosis prophylaxis.

With the preemptive use of ENPT for esophagectomies, which we implement in the form of preemptive active reflux drainage, we have frequently seen the positive benefit of the active elimination of biliary digestive secretions on anastomosis healing.

Endoscopic negative pressure therapy is suitable for the treatment of duodenal defects during postoperative management of complications, as well as for treating perforations. The main key to successfully treating duodenal defects, in addition to the defect closure itself, lies in the effective drainage of duodenal digestive secretions directed into the intraluminal space. When this drainage technique works effectively, wound contamination is stopped and the defects can heal.

The preemptive treatment approach of using active negative pressure drains can also be applied to operations in the duodenum. The underlying treatment principle is simple: Instead of passive overflow drainage, which never achieves complete drainage of secretions, negative pressure therapy actively eliminates digestive secretions. Instead of a passive gastroduodenal drainage tube, we use a drainage catheter equipped with an open-pore film to which suction can be applied.

We also used this simple therapeutic approach of active luminal drainage of secretions as an additional preemptive treatment measure in our case to ensure the healing of the duodenal suture.

Here, we present the method of preemptive intraluminal endoscopic negative pressure therapy (PINT) of the duodenum and outline the further course of treatment.

## Materials and methods

The aim of the PINT method is to evacuate the duodenal digestive secretions from the duodenal lumen and keep them away from the duodenal suture during the first vulnerable phase of wound healing.

A few hours after the surgical procedure, we initiated PINT of the duodenum to prevent duodenal suture insufficiency, covering the perforation defect and draining the bile secretions using a double-lumen open-pore film drain (dOFD). Negative pressure can be applied to one lumen of the tube, which is used to suction secretions. The other lumen corresponds to a feeding tube through which patients can simultaneously be given enteral nutrition while negative pressure is being applied.

**Fig. 2:**
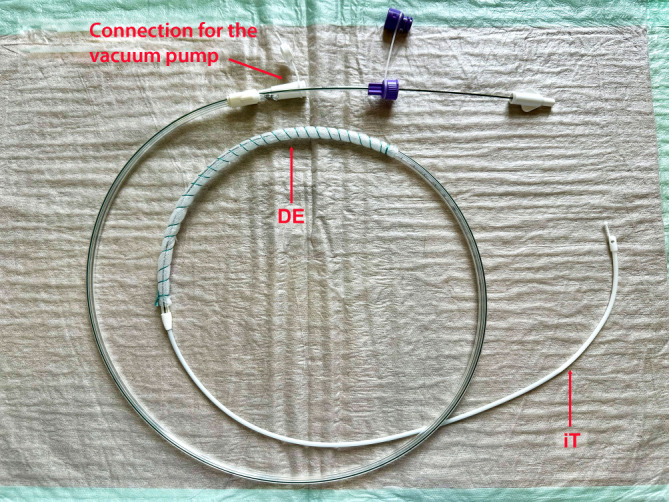
Double-lumen open-pore film drain: drainage element (*DE*) with film-wrapped gastric section and feeding tube (*iT*)

The dOFD was made using a Trelumina feeding tube (Freka® Trelumina, Fresenius SE & Co. KGaA, Bad Homburg, Germany) and the open-pore drainage film (Suprasorb® CNP drainage film, Lohmann & Rauscher International GmbH & Co. KG, Rengsdorf, Germany). The drainage element consisted of the gastric drainage section of the tube wrapped with the thin film over a total length of 25 cm. To secure the drainage film, we wrapped it with polyester thread (Mersilene®, Ethicon Deutschland Johnson & Johnson, Norderstedt, Germany). The tube’s ventilation section was closed with a clamp; it is not needed (Fig. 2).

### Specifications

**Fig. 3:**
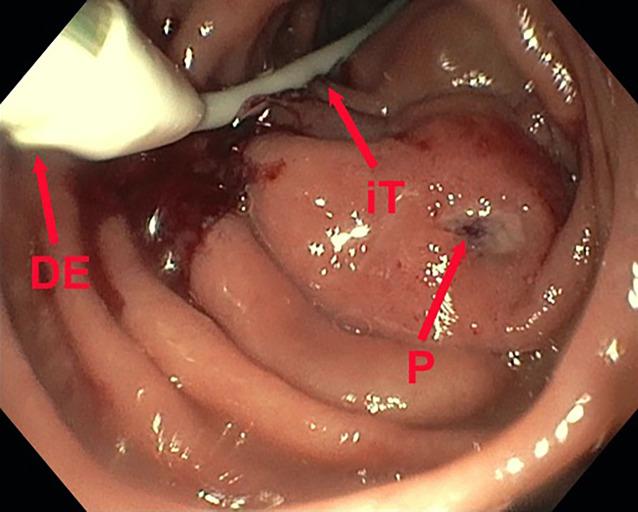
Placement maneuver for the double-lumen open-pore film drain. Feeding tube (*iT*), transition to the film-wrapped segment (*DE*), sutured perforation defect (*P*) of the nephrostomy catheter with perifocal edema and local signs of inflammation

The dOFD has a diameter of only 6 mm. Transnasal insertion was performed under endoscopic view using the same technique as with a gastroduodenal drainage tube. The intestinal feeding section of the dOFD was endoscopically advanced along the pylorus into the duodenum, and the dOFD was further advanced and placed deep into the duodenum with the open-pore film segment covering the defect region (Fig. 3).

### Specifications

A continuous negative pressure of −125 mm Hg was applied to the drainage element with an electronic negative pressure pump (Suprasorb® CNP endo Therapy Unit, Lohmann & Rauscher International GmbH & Co. KG, Rengsdorf, Germany).

By applying negative pressure, the duodenal secretions are permanently suctioned, the duodenal lumen collapses and is decompressed.

Through the integrated feeding tube that is located far distally from the application of negative pressure, intestinal feeding can occur at the same time as negative pressure is applied. Starting on the first postoperative day, the patient received tube feeding that was rapidly increased to a caloric intake of 2000 kcal. Supportive parenteral nutrition was not required. To keep the oral mucosa moist, the patient was allowed to drink water in sips; this was also suctioned.

According to our algorithm for ENPT, we performed an endoscopic assessment of the internal wound situation with a drain change of the dOFD every 3–4 days. An endoscopic assessment of the intraluminal suture conditions was conducted to determine whether the therapy would be ended or continued. For the endoscopy, we used CO_2_ examination gas.

The perioperatively initiated intravenous antibiotic therapy was continued.

## Results

**Fig. 4:**
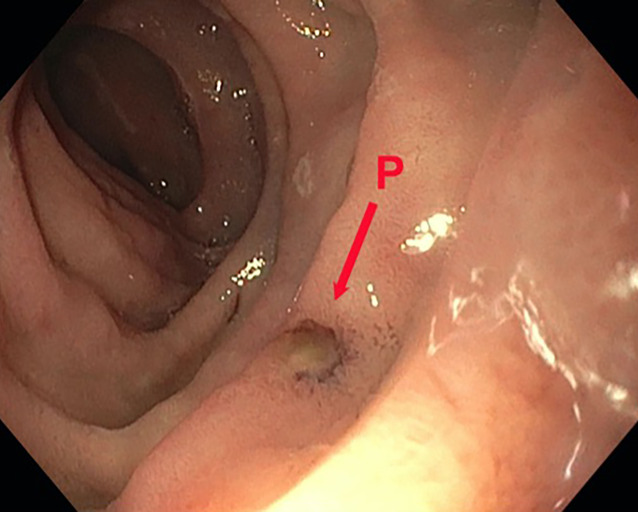
Perforation defect (*P*) on postoperative day 4, drain change with inspection of the internal wound. There is no sign of bile imbibition in the wound; the biliary secretions have been completely suctioned off. The perforation site does not appear inflamed and has a minimal fibrin coating; no defect detectable

In the first endoscopic assessment after 3 days, normal, non-inflamed wound conditions were observed with a slight fibrin coating on the suture, without further signs of inflammation and no defect. Perfusion was good. The initial perifocal edema was regressing. The dOFD was not dislodged and was located with the drainage element in the duodenum. A new dOFD was placed endoscopically without any problems, and the preemptive therapy was continued for a second period (Fig. 4).

### Specifications

The follow-up examination after an additional 3 days showed a non-inflamed internal duodenal wound with no signs of local infection.

**Fig. 5:**
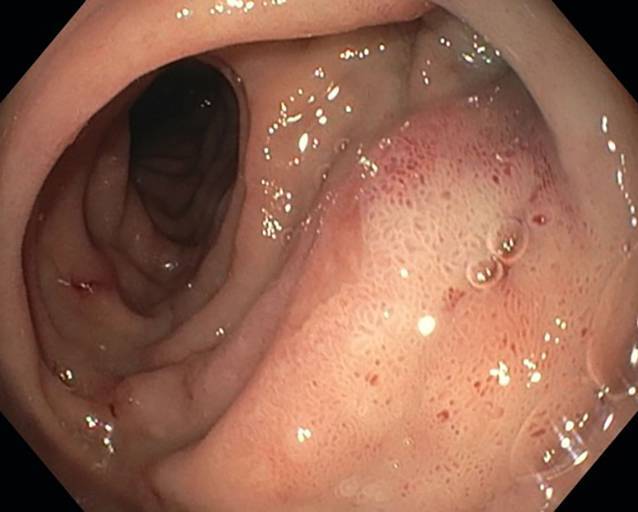
Termination of negative pressure therapy after 6 days on postoperative day 7, the perforation site has healed, only mild superficial signs of inflammation can still be seen

The preemptive negative pressure therapy was ended after a total of 6 days (Fig. 5).

### Specifications

In the final follow-up CT scan, no free intra-abdominal air, no evidence of an abscess or retention of secretions was found. The return to a normal oral diet proceeded over a few days progressing through liquids and pureed foods without any problems.

Summary of PINT: Insertion on the day of surgery, change on postoperative day 4, termination on postoperative day 7. Total treatment duration of 6 days, enteral nutrition via feeding tube.

**Fig. 6:**
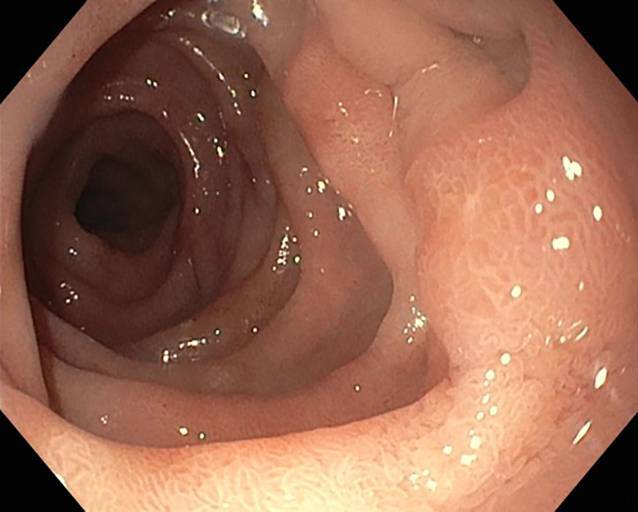
Perforation defect 5 weeks after the event with complete healing; no stenosis is observed

In an additional follow-up gastroscopy 5 weeks after the event, the duodenal perforation defect appeared to be completely healed (Fig. 6).

## Discussion

Using a case study of an iatrogenic duodenal perforation, we demonstrate how PINT can be applied preemptively in the duodenum in addition to surgical therapy. We demonstrate the technique with an open-pore film drainage tube, which includes an integrated feeding tube [8]. The healing of the sutured duodenal defect was unproblematic. We have not observed any complications related to the therapy.

The defect closure in the duodenum was performed surgically in our patient. Perforation defects can also be closed with endoscopic procedures, such as by using an over-the-scope clip (OTSC; [2]). Endoscopic therapy can always be considered when injuries occur from the luminal side. In such cases, closure via a luminal approach may also be indicated using an endoscopic method. It is a different situation when, as for our patient, a perforation was caused by a puncture from the extraluminal side of the duodenum. For such an injury pattern, it must be surgically evaluated whether or not other relevant extraluminal structures have been injured or whether there is an extraluminal infection. Endoscopic closure proceeding from the lumen alone may not be sufficient.

The preemptive application of negative pressure therapy developed from therapeutic ENPT. For esophageal defects, ENPT can now be regarded as the method of choice with a high success rate [9]. Open-pore negative pressure drains to which negative pressure can be applied via an electronic pump are used in intracavitary and intraluminal areas [3]. Two commercial drainage systems equipped with open-pore polyurethane foams are currently commercially available for the upper gastrointestinal tract (Eso-Sponge®; B. Braun SE, Melsungen, Germany; Suprasorb® CNP endo Oral and Oral‑N with Suprasorb® CNP endo Therapy Unit, Lohmann & Rauscher International GmbH & Co. KG, Rengsdorf, Germany). Open-pore negative pressure drains can also be constructed with a thin drainage film (Suprasorb® CNP Drainage Film, Lohmann & Rauscher International GmbH & Co. KG, Rengsdorf, Germany). This drain type we developed is of small caliber (diameter 4–6 mm) so that placement through small openings and transnasal placement are also possible [3, 8]. The tube can be equipped with a feeding tube, which allows for simultaneous nutrition. In addition to their use for esophageal defects, the indication spectrum of ENPT with these novel film-based drain types is extraordinarily broad [5, 10].

Moreover, ENPT is also suitable for the treatment of duodenal defects [7, 11]. We have been using it in the duodenum since 2010. Both polyurethane foam-based and film-based drains are used [7]. Of the two surgical treatment principles (defect closure and secretion drainage) in the duodenum, it is the drainage of duodenal digestive secretions toward the duodenal lumen that is particularly important. Bile can be effectively and easily drained toward the lumen using open-pore negative pressure drains. With ENPT, lumen-directed active elimination is facilitated. This prevents enzymatic contamination of the internal wound [7, 8, 11]. For PINT, the intraluminal variant of ENPT is used. The drains are placed intraluminally at the level of the treatment area with the drainage element composed of sponge or film. We use the method in selected cases when eliminating digestive secretions in the early postoperative healing phase is necessary for a beneficial effect on internal wound healing. In many cases, we were able to observe this on various anastomoses and thus since 2017 we have routinely implemented the procedure as a safety concept for esophageal resections at our institution. We use the presented film-based, double-lumen drain type with integrated feeding tube for this as well [6].

The drainage of gastric and duodenal secretions with passive drainage tubes does not achieve complete emptying of the stomach or duodenum. This observation can be confirmed from personal experience by any endoscopist who examines such patients. For active drainage, a complete elimination of the secretions can be achieved using open-pore drains made of polyurethane foam or drainage film.

We used a small-caliber open-pore film drain. This thin drain has the advantage that it can be easily introduced transnasally like a conventional gastroduodenal drainage tube. The passage along the pylorus into the duodenal lumen poses no difficulties either. From numerous applications with foam drains we know that the passage of a voluminous polyurethane foam body through the pylorus can occasionally be challenging.

Endoscopic negative pressure therapy with foam-based drains has already been used preemptively also after complex endoscopic interventions in the duodenum and esophagus. Hochberger et al. proposed the preemptive use of ENPT to prevent secondary perforation after endoscopic resection of large duodenal polyps [4]. Recently, Blasberg et al. reported on prophylactic use in the esophagus for extensive submucosal dissection of a superficial esophageal carcinoma [1].

All open-pore drains can become clogged. They then lose their suction effect and become ineffective. For this reason, a regular drain change with internal wound inspection is necessary. Our endoscopic examination intervals were very short, which led to an increased number of endoscopies that may pose a risk to the patient. An examination interval of 3–4 days, twice weekly, has proven effective for ENPT in our experience. In our case, this did not cause any complications.

The fact that gradually returning to an oral diet only occurs after the removal of the negative pressure drain could be considered a disadvantage. However, we were able to show that when using a double-lumen drain, immediate enteral nutrition through the feeding tube can occur at the same time as negative pressure therapy immediately after surgery.

## Conclusion

We report on the preemptive use of the ENPT as PINT in the surgical closure of an iatrogenic duodenal perforation. This new method aims to support the healing of the duodenal wound in the vulnerable first phase. For this purpose, the duodenal digestive secretions are eliminated postoperatively with an active negative pressure drain. The small-caliber open-pore double-lumen film drain used is equipped with a feeding tube. Enteral feeding can take place at the same time as the preemptive ENPT.

Additional studies are needed to evaluate the future value of PINT.

## Abbreviations

dOFD: Double-lumen open-pore film drain
ENPT: Endoscopic negative pressure therapy
PINT: Preemptive intraluminal negative pressure therapy

## Declarations

For this article, no studies on humans or animals were conducted by the authors. All studies mentioned were in accordance with the ethical standards indicated in each case. For images or other information in the manuscript that could identify the patient(s), a written consent form is available from them and/or their legal representatives.

The supplement containing this article is not sponsored by industry.
